# Theory of optimising radiation protection among diagnostic radiographers depicted as a model

**DOI:** 10.4102/hsag.v29i0.2730

**Published:** 2024-09-30

**Authors:** Shantel Lewis, Charlene Downing, Christopher M. Hayre

**Affiliations:** 1 Department of Medical Imaging and Radiation Sciences, Faculty of Health Sciences, University of Johannesburg, Johannesburg, South Africa; 2 Department of Nursing, Faculty of Health Sciences, University of Johannesburg, Johannesburg, South Africa; 3 Department of Medical Imaging, Faculty of Health, University of Canberra, Canberra, Australia

**Keywords:** ALARA, justification, patient care, optimisation, theory-generating

## Abstract

**Background:**

When radiation protection practices are suboptimal, it becomes necessary to take additional steps to optimise practices.

**Aim:**

Therefore, this study aimed to develop a model to facilitate radiation protection among diagnostic radiographers.

**Setting:**

The study was conducted in South Africa.

**Methods:**

A theory-generating design consisting of three steps was used to develop the model: (1) the central concept was identified, defined and classified; (2) relationship statements were created and (3) the model was developed, described and evaluated.

**Results:**

The model was premised on the central concept of facilitating a transformative radiation protection environment and occurs in four phases: (1) relationship, (2) working, (3) termination and (4) independent phases. The radiography manager and radiographer enter the relationship phase of the model because of discord regarding radiation protection in their department and work together to optimise radiation protection. The model’s purpose, assumptions, context, structure and process are described.

**Conclusion:**

A model to facilitate radiation protection among radiographers was conceptualised to optimise radiation protection. The model details the steps the radiography manager and radiographer take to co-create optimal radiation protection practices. Radiation protection compliance among radiographers is paramount to ethical radiography practice, and the model provides a guide to optimise radiation protection.

**Contribution:**

In any radiography department where radiation protection may be lacking, the model provides a road map of possibilities for change. Ultimately, radiation protection compliance improves patient care and outcomes.

## Introduction

Ionising radiation in medicine is the largest artificial source of radiation, compelling a careful balance between beneficence and maleficence of any exposure (Cho et al. 2018). The principles of justification, optimisation and dose limits guide radiation protection, and radiographers play a crucial role in ensuring these principles (Makanjee & Engel-Hills 2018). A radiographer is a healthcare professional providing medical imaging services. A radiographer in South Africa (SA) must be registered with the Health Professions Council of South Africa (HPCSA) in terms of the *Health Professions Act* (SA 2020), practising within the scope of practice (HPCSA 2020:1–5). Radiographers may register in the categories of diagnosis, therapy, nuclear medicine or ultrasound. Diagnostic radiographers work in diagnostic imaging where ionising and non-ionising radiation are used to produce and record anatomy and physiological functions. Diagnostic radiographers integrate patient history and clinical data in decision-making to produce high-quality images (HPCSA 2020:1–5). Diagnostic radiographers (*hereafter referred to as radiographers*), therefore, review the patient’s clinical history to determine whether the imaging examination requested is justified and to select the imaging protocols and projections that will demonstrate the required area optimally. Throughout the process, the radiographer must weigh the benefit of every decision against the risk of exposure to ionising radiation, keeping in mind the principles of radiation protection (Makanjee & Engel-Hills 2018).

However, previous studies evince radiographers’ non-compliant and suboptimal radiation protection awareness and practices (Abo El Aish, Abuqamer & Alajerami 2022; Hawarihewa et al. 2022; Hayre 2016; Maina, Motto & Hazell 2020; Yamashina et al. 2022). Literature provides evidence of incorrect protocols, imaging of wrong anatomical areas or sides and exposure creep, where 26% of images reviewed were obtained using milliampere-seconds (mAs) greater than the acceptable value (Farzanegan et al. 2020; Tarkiainen et al. 2020; Warren-Forward et al. 2007). These transgressions were related to human and procedural errors. As radiographers are crucial to ensuring radiation protection, deficient radiation protection necessitates enhancing radiation protection compliance among radiographers. Therefore, this study conceptualised a model to facilitate radiation protection among diagnostic radiographers. A model is the formal, symbolic, schematic depiction of relationships between phenomena and reality conceptualised through empiric knowledge development. Models use words, symbols or graphic diagrams to represent the relationship of concepts within a theory (Chinn, Kramer & Sitzman 2022). Model development is not common in medical imaging research, whereas much more literature is available in nursing research. Therefore, this study presents an opportunity to review the theory generation research design application within the context of medical imaging.

## Research methods and design

A theory-generating research design, based on Chinn and Kramer’s (2015, 2018) empiric knowledge development, was used in this study to develop, describe and evaluate a model that facilitates radiation protection among radiographers. The three steps of theory generation are discussed in the following sections.

### Step 1: Identifying, defining and classifying the central concepts

Concepts framing the model need to be identified and then defined and classified. Concepts convey the meaning of theory; therefore, explicit definitions of the concepts are required to understand the theory (Chinn et al. 2022). Concepts in the current study were identified from the findings of an explanatory, sequential, mixed-method study set in South Africa (Lewis, Downing & Hayre 2022a, 2022b, 2022c, 2023). Table 1 provides a summary of the three phases of the explanatory, sequential, mixed-method study.

**TABLE 1:** Summary of the three phases of the explanatory, sequential, mixed-method study (Lewis et al. 2022a, 2022b, 2022c, 2023).

Population (6552 diagnostic radiographers)	Sampling purposive	Respondents and participants	Data collection	Data analysis	Reliability and validity or trustworthiness	Findings
Phase 1	Facebook WhatsApp radiography seminar and departments	417 (6.4% response rate)	Online questionnaire	Descriptive and inferential statistics Correlation analysis	Reliability: Cronbach’s alpha; Construct validity: Factor analysis; Pilot study	Attitudes, subjective norms and perceived behavioural control were high.
Phase 2	27 respondents from phase one agreed to participate in phase 2.	13 (representing eight provinces in South Africa)	In-depth interviews	Thematic analysis: Braun and Clarke (2021:128–148): Data familiarisation, coding, initial theme generation, theme development and review, refining, defining and naming themes and writing up.	Credibility: Triangulating interview data, notes of the interviews and literature. Dependability and transferability: Detailed description of the research methodology. Confirmability: Audit trail detailing data collection, analysis and interpretation together with reflexivity.	Radiation protection knowledge is good, but suboptimal practices are attributed to a lackadaisical attitude and patient and work factors. Compliance was a personal choice: Practising RP depends on the individual radiographer and their diligence. Greater RP compliance for paediatrics, pregnant and oncology patients. Influenced by their colleague’s practice, the culture in the department and radiologists. Reasons for non-compliance: Lack of support from radiology management. The medical team did not value their opinion. Radiographer shortages, limited X-ray rooms, time spent locating RP equipment, being rushed with trauma or challenging patients to complete exam results in non-compliance. Use of RP equipment varied. Established protocols were not enforced. Inadequate training – failure to understand equipment optimally. Influenced by the culture in the department.
Phase 3	Snowballing Radiography managers	8 (Participants’ roles: 1 deputy director role, 3 assistant directors, remaining managing radiographers representing three of the nine provinces in South Africa)	2 focus group interviews			To ensure conscious and consistent compliance to radiation protection, the following were suggested: Raised awareness of radiation protection Continuous maintenance of equipment Standardised national protocols and radiography organisational structures. Holistic integration of radiation protection involving management and frequent compliance monitoring.

### Step 2: Construction of relationship statements

The concepts from step one were written into relationship statements. Relationship statements interrelate concepts of theory and constitute the theory’s ‘substance and form’ (Chinn et al. 2022). Therefore, the relationship statements demonstrate links between the central concepts, creating a preliminary conceptual model (Lewis, Downing & Hayre 2024).

### Step 3: Description and evaluation of the model

In step three, the model is developed, described and evaluated. The conceptual model for the current study was designed using Chinn and Kramer’s (2015, 2018) empiric knowledge development process. The theory was structured and contextualised, and theoretical relationships were developed and tested. The model’s structure was described and evaluated according to the five model evaluation components of clarity, simplicity, generality, accessibility and importance to ensure the developed model was based on a theory. The model was evaluated by nine experts who were purposively sampled for their expertise in theory and model development. The model was presented by the first author via MS Teams to the nine experts. At the end of the presentation, the experts completed an online questionnaire that asked: ‘How clear is the model?’ ‘How simple is the model?’ ‘How general is the model?’ ‘How accessible is the model?’ and ‘How important is the model?’. The questionnaire responses were allocated a pseudonym for confidentiality (e.g. E1 – expert 1).

### Ethical considerations

The study received ethical approval from a higher education research ethics committee (REC-01-28-2019). The study information letter, detailing the aim of the model, the model evaluation process, the right to withdraw from participation, the confidentiality and privacy of their participation and the researcher’s details for questions was shared via email with the model experts. The experts signed the consent form electronically and returned it via email. The signed consent forms were then stored on Google Drive, accessible to the first author through a password. All collected data were de-identified and securely stored.

## Results and discussion

The model was evaluated by nine experts, and their demographics are presented in Table 2.

**TABLE 2:** Model experts’ demographics.

Expert	Qualification	Industry	Designation	Years of clinical experience	Years of academic experience	Years of experience in model development
1	Doctorate or PhD	Academic	Senior lecturer	4	21	12
2	Doctorate or PhD second year	Academic	Lecturer	10	9	4
3	Doctorate or PhD	Academic	Senior lecturer	28	9	2
4	Doctorate or PhD	Compliance and research	Nurse manager	28	15	4
5	Doctorate or PhD	Academic	Lecturer	9	16	3
6	Doctorate or PhD sixth year	Academic	Lecturer	20	18	5
7	Doctorate or PhD	Academic	Lecturer	3	9	2
8	Doctorate or PhD	Clinical nursing	Operational manager	22	5	5
9	Doctorate or PhD	Clinical nursing	Quality and Risk Coordinator or ED Nurse	7	10	4

Six of the experts were from the medical imaging and radiation sciences field and provided 74 years of clinical experience. The model evaluation results are presented under step 3.

### Step 1: Identifying, defining and classifying the central concepts

The explanatory, sequential, mixed-method study (Lewis et al. 2022a, 2022b, 2022c, 2023) revealed radiation protection practices to be suboptimal, with evidence of non-implementation of radiation protection in some instances. Radiographers’ attitudes, subjective norms and perceived behavioural control were high, but compliance was a personal choice, and poor compliance was observed despite good knowledge about the basic principles of radiation protection. Radiography managers also observed deficient radiation protection practices. Radiographers’ practice is influenced by their managers, colleagues, radiologists, the healthcare team and patients. Hence, to optimise radiation protection practices, the support of these influencers is necessary. Radiographers’ confidence and control in performing radiation protection are also affected by available resources, radiation protection policies and their organisation’s safety culture. To optimise radiation protection practices, all radiographers should have positive attitudes towards it, and a multifactorial approach inclusive of the individual radiographer and the environment in which they function is necessary to implement and optimise radiation protection. These findings led to the central concept of ‘facilitating a transformative radiation protection environment’. The central concept is defined as the process of helping to promote a total internal and external context that fosters change in implementing and optimising the safety of X-ray exposure while considering the benefits and risks of the X-ray exposure (Lewis et al. 2024). The central concept was classified using Dickoff, James and Wiedenbach’s (1968) list of survey questions:

1. *Who is the agent?*
  In the current study context, who is responsible for facilitating a transformative radiation protection environment? The radiography manager (a term referring to the radiography leader, supervisor, in charge or head radiographer who is the radiographer who manages radiographers in a radiography department) is responsible for facilitating a transformative radiation protection environment. The radiography manager’s duties include ensuring compliance with health and safety and radiation protection legislation.
2. *Who is the recipient?*
  In the current study’s context, who will receive the outcomes of facilitating a transformative radiation protection environment? The radiographer is the recipient.
3. *What are the dynamics?*
  Here, the question is, ‘What is the energy source for the activity?’ The energy driving facilitating a transformative radiation protection environment is the need to implement and optimise radiation protection among radiographers driven by step one’s findings.
4. *What is the procedure?*
  Here, the question is, ‘What is the activity’s guiding procedure, protocol or technique?’ As the agent, the radiography manager engages with internal and external stakeholders in the radiography department. Each stakeholder has a vital role in supporting the implementation and optimisation of radiation protection. Therefore, the radiography manager liaises with these various stakeholders, as indicated in Table 3, to ensure a transformative radiation protection environment.
5. *What is the context?*
  The context of the study is the radiography department, which is managed by the radiography manager and where the radiographer works. A radiography department exists within the healthcare system.
6. *What is the terminus?*
  Implementing and optimising radiation protection among radiographers is the endpoint of facilitating a transformative radiation protection environment.

**TABLE 3:** Stakeholders in radiation protection.

Stakeholders	Responsibilities within radiation protection (RP)	Related to suggested ways to foster RP as determined through the mixed method study (Lewis et al. 2022a, 2022b, 2022c)
**External**		
Radiation Control	Confirms compliance and monitors ionisation radiation	A national radiation dose registry. A standardised national protocol on RP. Accountability. Frequent compliance monitoring. Increase RP awareness – healthcare team, patient and public. Re-engineering RP. Regular safety conversations, awareness and peer reviews. Provide support to radiography managers and radiographers to mitigate RP non-compliance since radiography managers misunderstand their role as managers and the diminished stature of the radiographer.
Chief Executive Officer	Drives the organisational culture	Linking RP to the organisation’s objectives. Collaborating with different role players. Improving communication between hospital management, doctors, radiography managers and radiographers at different institutions. Regular safety conversations. Awareness, peer reviews. Increasing RP awareness – healthcare team, patient and public. Aid in improving the stature of the radiographer so they are respected and supported concerning RP.
Chief Finance Officer	Approves finance to support RP initiatives	Well-resourced: Equipment maintained, functioning optimally. Increase RP awareness – healthcare team, patient and public.
Procurement	Secures resources to support RP	Well resourced. Increase RP awareness.
Information and communications technology	Information technology that allows patients to be registered and scheduled, image storage and retrieval, DICOM data and radiologist reports	Well resourced: Equipment maintained, functioning optimally. Increase RP awareness – healthcare team, patient and public.
Radiography managers from other institutions	Communicates RP policies, protocols, best practices, challenges and concerns.	Improving communication between hospital management, doctors, radiography managers and radiographers from different institutions.
Healthcare team		
Doctor Nurses Speech therapists Dentists Chiropractor Physiotherapist	Refers patients for X-rays. Completes the X-ray request form with the correct patient details, clinical history and requested examination.	Increase RP awareness. Regular safety conversations. Awareness, peer reviews. Teamwork and communication regarding radiographers’ diminished stature and RP attitude. - -
Patient	Provides a complete and clear clinical history	Increase RP awareness and knowledge.
**Internal**		
Administrators	Capture patients’ data and examinations requested, schedule patients for examinations, and return completed X-ray examinations with matched radiologist reports to the correct patient.	Accountability. Frequent compliance monitoring. Increase RP awareness. Regular safety conversations. Awareness, peer reviews.
Radiographers	Responsible for X-raying the patient while adhering to the ALARA, justification, optimisation and dose limit principles.	Changing mindsets and small practices. Changes to improve RP compliance. Ethical, consistent and conscious compliance. Radiographers empowered through education: Latest technology, communication skills and patient care. Accountability. Frequent compliance monitoring. Regular safety conversations. Awareness, peer reviews. Support from hospital and radiography management to mitigate conditions hindering compliance.
Student radiographers	Responsible for X-raying the patient while adhering to the ALARA, justification, optimisation and dose limit principles under supervision.	Accountability. Frequent compliance monitoring. Regular safety conversations. Awareness, peer reviews. Supervision.
Patient	Understands and listens to the instructions provided.	Improve patient’s knowledge of RP. Improve radiographers’ stature.
Radiologist	Responsible for approving and performing radiographic examinations of the patient while adhering to the ALARA, justification, optimisation and dose limit principles. Provides a specialist report.	Accountability. Frequent compliance monitoring. Regular safety conversations. Awareness, peer reviews.
Nurse in the radiography department	Assists the radiologist in radiographic examinations.	Accountability. Frequent compliance monitoring. Regular safety conversations. Awareness, peer reviews.
Porters	Transport patients from the wards and outpatient departments to and from the radiology department. They are responsible for bringing the correct patient to the X-ray department with the correct patient documentation and transportation device.	Accountability. Frequent compliance monitoring. Regular safety conversations. Awareness, peer reviews.
Radiography manager	Responsible for the operation of the entire radiography department. Drives department safety culture.	Recognises the total internal and external context within which the radiographer practises RP. Complex cultural and social conditions, interrelationships, social relationships and power relations. A radiographer is a whole being who has motivations and is affected by internal and external factors, culture and beliefs. Radiographer’s inner discomfort and perplexity when their worldview is challenged or a disorientating dilemma will engage in critical reflection and discourse for beneficial, complete, dramatic and marked change to grow, develop, perform and optimise daily radiation protection activities. Responsibility to help by supporting the radiographer through dynamic, unique processes to re-create radiation protection practices. Diminished stature of the radiographer. Well resourced: Equipment maintained, functioning optimally. Linking RP to the organisation’s objectives. Collaboration with different role players. Improving communication between hospital management, doctors, radiography managers and radiographers from different institutions. Regular safety conversations. Awareness, peer reviews. Increase RP awareness – healthcare team, patient and public. Radiographers getting respect. Radiographers are empowered through education: the latest technology, communication skills and patient care.
Practice manager (private)	Responsible for the management of the radiography department.	Improved the stature of the radiographer. Well resourced: Equipment maintained, functioning optimally.

### Step 2: Construction of relationship statements

The relationship statements guiding the model to facilitate radiation protection among radiographers are:

- The radiography manager facilitates a transformative radiation protection environment that enables the implementation and optimisation of radiation protection.
- The radiography manager and the radiographer, disoriented by the dilemma of non-implementation or suboptimal radiation protection practices, undertake the dynamic process of critical reflection and discourse about applying radiation protection measures. The radiography manager encourages the radiographer to reflect on their own radiation protection practice and engages in change dialogues to optimise radiation protection.
- The radiography manager engages with all radiation protection stakeholders to explain their roles in radiation protection and how their compliance will affect radiographers implementing and optimising radiation protection.
- The radiographer is empowered by a supportive environment and implements and optimises radiation protection, and the radiography manager and radiographer assess the radiation protection practices.
- When optimal radiation protection practices are implemented, the radiographer can continue independently.

### Step 3: Description and evaluation of the model

The model is described according to its purpose, assumptions, context, structure and process. The purpose of the model is to provide a theoretical framework of reference to facilitate radiation protection among radiographers.

A model’s assumptions guide theoretic interpretations by making the meaning of the model explicit (Chinn et al. 2022). The meta-theoretical assumptions of the study were situated within the theory of planned behaviour considering the person, environment, health and caring (Ajzen 1985). Based on the theory of planned behaviour, it is assumed that radiography managers and radiographers consider available information and the implications of their actions for radiation protection’s implementation and optimisation in the radiography department. The radiography manager and radiographer will act according to their intention and acknowledge their different abilities to control their radiation protection actions. The success of radiation protection implementation and optimisation is incumbent on the radiography manager and radiographer, who possess the required information, skills and abilities. They also have willpower and control over their emotions and compulsions. In addition, success depends on the various stakeholders acting in accordance with enabling compliance. The radiography manager has the experience and expertise to lead the radiography department, while the radiographer has completed their radiography qualification and is registered with the legislated body.

**FIGURE 1:**
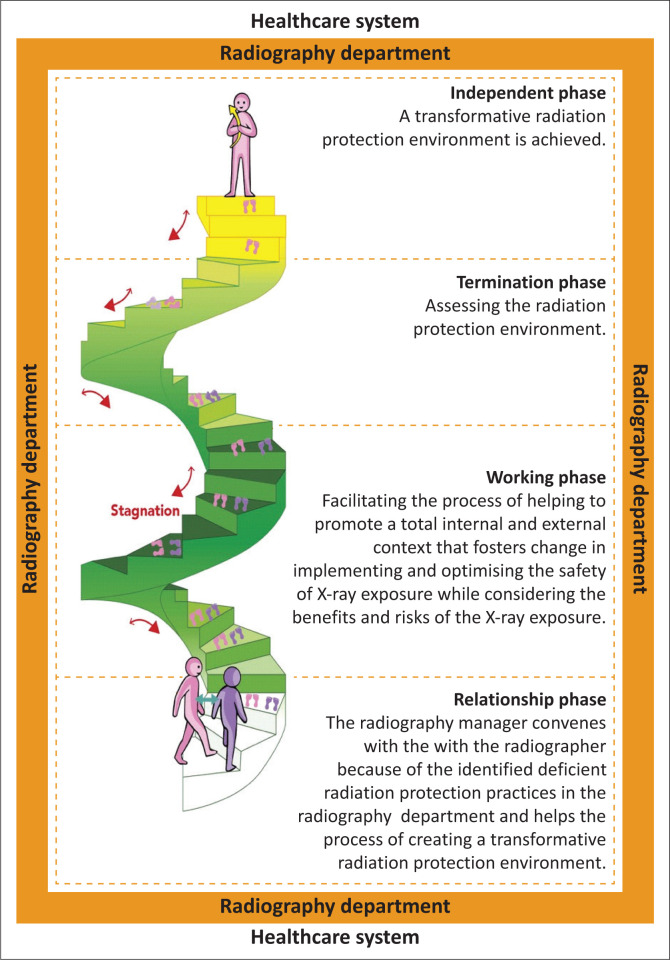
Model to facilitate radiation protection among radiographers.

Health, in the model, considers the radiography manager, the radiographer, all stakeholders directly involved in the X-ray exam and the public. As radiation protection is entrenched in the radiography manager and radiographers’ moral and ethical practice (HPCSA 2008), a contrary practice may affect their harmony of being an integrated whole person. This disorientation is the antecedent of transformation. An environment supportive of radiation protection transformation is thus required to ensure harmony is restored. The radiography managers’ and radiographers’ health are also influenced by their environment; they will be comfortable and harmonious if the environment supports and mirrors their personal radiation protection beliefs and practices. In the model, caring is expressed as the radiography manager and radiographer’s competence in implementing and optimising radiation protection. It entails limiting patients’ exposure to ionising radiation and actuating person-centred care and caring during the X-ray exam (Atutornu & Hayre 2020). The model context is the radiography department. The model’s structure and process are depicted in Figure 1.

The model’s structure is explained through basic structures, colours and symbols. The model is structured using a spiral stairway and two people who embark on the process of transforming the radiography department’s radiation protection practices, illustrated in Figure 1, through four phases: relationship, working, termination and independent phases. The context of the model is illustrated by the model’s borders representing the radiography department that operates within the healthcare system; it is a closed system. The colour selected for the border was orange as it signifies creativity and simulation. Orange is about togetherness and sharing, respect for opinions and choice and attracting a deep feeling of support and loyalty (McLeod 2016).

The facilitator in the model is the radiography manager who possesses the required leadership, supervisory, planning, interpersonal and organisational skills to mobilise necessary resources and communicate with stakeholders to ensure quality radiography services and is shown as the violet figure on the right at the stairway’s base. The image used to represent the radiography manager is gender neutral, representing all ages, races and cultures so that radiography managers could envision themselves in the model, irrespective of their gender preference, age, race or culture. Violet represents being supportive, inspiring, motivating and uplifting. The recipient in the model is the radiographer, depicted as the pink figure on the left of the stairway’s base. Pink represents energy that increases creativity and motivation. The radiographer will need to harness creativity and be motivated to change to implement or optimise radiation protection.

The blue bidirectional arrows note the relationship between the radiography manager and the radiographer; blue represents the qualities of serenity, stability, inspiration and wisdom that exist in the relationship between the radiography manager and radiographer. The footprints on the spiral stairway indicate the radiography manager and radiographer’s interaction and movement as they embark on the transformation journey through dialogue, critical reflection and gaining knowledge. Because of the dynamic process, the radiography manager and/or radiographer may decide to stop the process and re-enter the journey if there is discordance between their expectation and the actuality, as shown by the red arrows; red signifies the desire to take action to improve. The arrow is bilateral, reflecting movement within the dynamic process. A spiral stairway was used because stairs represent progress, transcendence or ascension (Cirlot et al. 2020). The spiral shape of the staircase represents the process of evolution, life and growth.

The relationship phase is shown at the base of the stairway, where the radiography manager and radiographer come together. The relationship phase is smaller than the other phases as the radiography manager and radiographer embark on the journey because of mutual understanding, represented in the model by fewer stairs. The relationship phase is made up of the white stairs; white reflects all colours of the spectrum and serves as a metaphor for the radiography manager and radiographer’s reflective process that brought them to this point. In addition, the colour white is achromatic, having no hue and can thus be perceived as pure, symbolising the radiography manager and radiographer’s pure intentions to protect all entities involved in an X-ray examination (Holtzschue 2017). White also means a new beginning, and as the radiography manager and radiographer are embarking on the new process, white was selected for the relationship phase (Cherry 2020).

The radiography manager and radiographer move to the working phase once they have agreed on the goal of the dynamic, transformative process on which they are about to embark. Most facilitation occurs during the working phase and is therefore presented as the dark green steps on the stairway. In this context, dark green is taken to mean growth (Cherry 2020). Thus, both the radiography manager and radiographer enter this phase, ready to grow and develop. A greater number of stairs represents the working phase because of the extensive work of engaging with and relying on multiple stakeholders to ensure the creation of a transformative radiation protection environment.

The success of the working phase is assessed in the termination phase. The termination phase is smaller than the working phase and is represented by the light green steps; light green indicates renewal and optimism (Cherry 2020). Once radiation protection is implemented and optimised, the radiography department experiences a renewal of attitudes and habits and is optimistic that the changes will prevail.

The independent phase is represented by the yellow steps of the uppermost part of the stairway; yellow, in this context, represents radiography managers’ and radiographers’ enlightenment in implementing and optimising radiation protection. The accumulated knowledge through the relationship, working and termination phases culminates in the radiography manager and radiographer being invested in radiation protection and ambitious and attentive to ensuring compliance. Continued learning and growth are the hallmarks of the transformative radiation protection environment, underpinned by critical reflection and change dialogues indicated by the radiographer holding an arrow at the top of the stairway. They also possess the intellect to make the personal choice to practise radiation protection.

The process of the model, from the relationship to the independent phases, is described. The four phases are dynamic, and movement may occur between and among the phases.

### Relationship phase

The relationship phase is the first phase of the model. It begins when either the radiography manager or radiographer – as whole beings who have motivations – experience inner discomfort and perplexity over the current radiation protection practices in the radiography department, coming together willing to recreate their radiography department’s radiation protection practices and culture (Lewis et al. 2024). There is an acknowledgement that multiple stakeholders are involved in ensuring radiation protection’s implementation and optimisation, and the environment consists of complex cultural and social conditions, interrelationships, social relationships and power relations that are affected by internal and external factors, culture and beliefs. As a facilitator, the radiography manager will help the radiographer by communicating with radiation protection stakeholders to make the dynamic process easier for the radiographer. The radiography manager will support the radiographer who embarks on critical self-reflection on radiation protection practices and change dialogues. The radiography manager will assist the radiographer in clearly outlining the dynamic process of creating a transformative radiation protection environment. Explaining the process enables the radiographer to understand that the process is dynamic and empowering to all stakeholders.

### Working phase

The working phase is the second phase of the model and begins with the radiography manager and radiographer meeting to analyse the discord between legislated radiation protection and radiation protection practices in the radiography department. The radiography manager asks the radiographer to spend some time critically self-reflecting on radiation protection practices and factors that impact and influence these practices. The radiography manager reflects on their role in radiation protection in the radiography department. The radiography manager and the radiographer also examine all the external and internal stakeholders influencing non-compliance with radiation protection in the department. The radiography manager and the radiographer take all aspects of their analysis into account and plan the process of creating a transformative radiation protection environment. A generic plan was created, shown in Table 4, using step one’s findings; however, individualised plans can be determined by the analysis undertaken in the radiography department.

**TABLE 4:** Radiography managers’ engagement during the model’s working phase.

Stakeholders	Purpose	Radiography manager engagement
**External stakeholders**		
External to the hospital or radiology department		
Radiation control or monitoring board	Ensure compliance and monitoring of ionising radiation	Standardised national protocol on radiation protection. Establish an individual national patient dose register. Frequent monitoring. Hold accountable deviant practices. National SWOT analyses to re-engineer radiation protection. Create radiation protection awareness among administrators, the healthcare team, patients and the public. Encourage regular safety conversations Conduct a peer review of radiation protection practices.
Radiography managers from other institutions or departments	Support by communication on the application of radiation protection policies, protocols, challenges and concerns	Application of radiation protection. Process of implementing and optimising radiation protection. Challenges. Radiation protection best practices.
External to the radiography department but within the hospital		
Chief executive officer of the institution where the radiography department is located	Drives the organisational culture	Made aware of their contribution to adhering to radiation protection principles and limiting all unnecessary exposure to the patient. Custodian of radiation equipment licences, as stipulated in the Code of Practice for Users of Medical X-ray Equipment. Include radiation protection in all the organisation’s objectives. Collaborate with all radiation protection stakeholders, creating awareness of radiation protection. Facilitate the improvement of communication between radiation protection stakeholders.
Chief finance officer, procurement and information and communications technology	Financial, resource and information technology support enabling radiation protection.	Engage with them on their contribution to adhering to radiation protection principles and limiting all unnecessary exposure to the patient. Importance of financing quality equipment. Role in supporting and enabling the radiographer.
The healthcare team	Requests X-ray examinations and assists the radiographer in providing imaging services remote to the radiography department	Engage with the healthcare team on their contribution to adhering to radiation protection principles and limiting all unnecessary exposure to the patient. Ensure accurate and correct X-ray requests. Ensure mobile X-ray requests meet specified criteria. Assist radiographers in imaging outside the radiography department.
Patient	The patient must be honest and provide the X-ray exam requestor with a correct and detailed clinical history	Share patients’ rights and responsibilities Communicate risks and benefits
**Internal stakeholders**		
Porters	Transport the correct patient with correct patient documentation in the proper transportation device.	Engage with the porters on their contribution to adhering to radiation protection principles and limiting all unnecessary exposure to the patient. Accurately complete the activities.
Administrators	Capture patients’ data and requested examinations, schedule patients for examinations, and return completed X-ray examinations with matched radiologists’ reports to the correct patient.	Engage with the administrators on their contribution to adhering to radiation protection principles and limiting all unnecessary exposure to the patient. Accurately complete the activities.
Patients requiring X-ray examinations	Understand their role in achieving optimal diagnostic imaging	Accurate history Disclose pregnancy Follow radiographers’ instructions
Nurse in the radiography department	Assists the radiologist in radiographic examinations	Engage with the radiography department’s nurse on their contribution to adhering to radiation protection principles and limiting all unnecessary exposure to the patient. Correct patient and correct examination Ensure radiation protection during specialised procedures
Radiologist	Approves and performs radiographic examinations. Provides a specialist report.	Engage with radiologists on their contribution to adhering to radiation protection principles and limiting all unnecessary exposure to the patient. Recommend suitable imaging examinations to limit exposure to X-rays. Provide support to ameliorate the radiographer’s diminished status.
Practice managers	Responsible for the management of the radiography department.	Engage with practice managers on their contribution to adhering to radiation protection principles and limiting all unnecessary exposure to the patient. Garner support
Student radiographers	Perform radiation protection supervised	Contribution to adhering to radiation protection principles and limiting all unnecessary exposure to the patient. Encourage the radiographer to supervise the student, aligned with the radiation protection culture of the radiography department.
Radiographer	Responsible for X-raying the patient while adhering to the ALARA, justification, optimisation, dose limit principles and code of practice.	Communicate support garnered from all radiation protection stakeholders. Have regular conversations about safety with stakeholders. Radiation protection is incumbent on the radiographer’s choice to practise. Discuss compliance deviations. Understand motivations. Radiation protection and safety aligned with the Code of Practice for Users of Medical X-ray Equipment. Appointing a quality control radiographer

ALARA; As Low as Reasonably Achievable; SWOT, strengths, weaknesses, opportunities, and threats.

During the working phase, the radiography manager acknowledges the radiographer’s challenges surrounding the lack of support from radiology management, their opinion of not being valued by the medical team, radiographer shortages, limited X-ray rooms, time spent locating radiation protection equipment and being rushed when attending to trauma or challenging patients to complete the exam. The radiography manager notifies the radiographer of the engagement activities undertaken with radiation protection stakeholders to address these challenges and support the radiographer (Table 4). Radiation protection compliance, fully supported by all radiation protection stakeholders, is now incumbent on the radiographer’s choice to practise radiation protection. The radiography manager reverts to the radiographer’s critical self-reflection on their radiation protection practices and discusses any compliance deviations.

The radiography manager compliments radiographers on their radiation protection knowledge and takes the opportunity to encourage the application of this knowledge. The radiography manager engages in dialogues supporting and encouraging radiographers always to be diligent and choose to implement and optimise radiation protection. The radiography manager engages with radiographers to understand their motivation to comply with radiation protection in certain patients. The radiography manager then harnesses these motivations to encourage radiographers to apply radiation protection consistently in practice.

The radiography manager builds a strong radiation protection culture by engaging in safety dialogues with all stakeholders. Radiographers are requested to monitor compliance in the radiography department and support students’ compliance. Any compliance deviations should be discussed through constructive change dialogues. The radiography manager supports radiographers by assisting in providing optimal training on radiography equipment they find challenging. The radiography manager reminds radiographers that radiation protection is an ethical requirement and their core practice. However, in acknowledging the radiographer’s motivations, the radiography manager introduces a radiation protection radiographer of the month, as selected by the radiography department. The radiation protection radiographer of the month shares their experiences of applying radiation protection in the radiography department to encourage the continued application of the practice. The award is also acknowledged in performance management reviews. The appointment of a quality control radiographer to monitor collimation, exposure factors and adherence to radiation protection may be considered.

The working phase addresses all the challenges identified through critical reflection to help and support radiographers in a transformative process of implementing and optimising radiation protection. At any time in the working phase, the radiography manager and radiographer may decide to move to the relationship phase and re-enter the working phase when ready. However, if the working phase addresses the identified radiation protection compliance challenges, the radiography manager and radiographer move to the termination phase.

### Termination phase

The termination phase is the third phase of the model, where the radiography manager and radiographer have addressed all the identified radiation protection compliance challenges and now need to assess whether the working phase was successful. The radiography department is considered successful when radiation protection is implemented and optimised by establishing a transformative radiation protection environment. The radiography manager and radiographer acknowledge that compliance from some stakeholders external to the institution where the radiography department is situated may be long term and not within the radiography manager’s control. Therefore, initiating engagement with external stakeholders will be considered an achievement in the process. The radiography manager and radiographer can say goodbye to the process, knowing that the transformative radiation protection environment is sustained. Radiation protection is inculcated in the radiography department and the institution’s culture. The depth and breadth of radiation protection knowledge are ingrained in the thoughts, words, actions, habits and attitudes of radiographers and all stakeholders. At any time, the radiography manager and radiographer may experience stagnation and re-enter the model’s working or relationship phase when they are ready. With the radiography department’s transformative radiation protection established, the radiography manager and radiographer enter the independent phase.

### Independent phase

The last phase in the model is the independent phase. The radiography manager and radiographer enter the independent phase when a sustainable, transformative radiation protection environment is established and functions without the radiography manager and radiographer guiding the process. Radiation protection has been optimised and supported by all stakeholders. The radiography manager and radiographer may experience stagnation in any model phase and re-enter the consecutive phase when ready. A transformative radiation protection environment is dynamic and fosters continual evolution incumbent on all stakeholders’ compliance.

Table 5 shows the areas of improvement suggested by the model experts. The model presented in this article encompasses the comments from the experts.

**TABLE 5:** Model experts’ evaluation of the model.

Criteria	Meaning	Evaluator comments	Action
Clarity	Refers to the understanding and conceptual consistency of the theory: Structural clarity: explicit organisation and interconnectedness of concepts Structural consistency: the consistent use of interconnecting structures Semantic clarity: the explicit or implicit definition of concepts Semantic consistency: the consistent use of the concepts expressing the intended meaning.	‘[*L*]egislation needs to be included in the model’. (Expert 1) ‘[*T*]o include a team approach as opposed to individual is recommended. Colour shading and terminus of the model should be reviewed.’ (Expert 2) ‘[*T*]he downward arrows bring the idea of regression and not stagnation.’ (Expert 4) (similar to Expert 7) ‘I’m not sure what the radiographer is holding in her hand? Or if there is significance in the manager having his hand in his pocket?’ (Expert 5)	Empirical findings in the study indicate that radiation protection legislation is provided, but compliance remains challenging. Therefore, the model to facilitate radiation protection was developed. The agent and recipient in the model are explained using Dickoff, James and Wiedenbach’s^20^ list of survey questions. Even though the model refers to the individual radiographer and radiography manager, the stakeholders are indicative of the teamwork and collective effort required to create a transformative radiation protection environment. The colour shading and terminus were revised. The arrows were amended. Radiographer and radiography manager images amended.
Simplicity	Minimal use of concepts and theoretical relationships to express understanding and the interrelationship of concepts.	‘The footprints in the working phase could be bigger and become smaller and smaller.’ (Expert 5) ‘The gender should be more gender neutral.’ (Expert 6) ‘Why are there 2 steps for working and termination and one for relationship and terminus?’ (Expert 7) ‘The staircase is very linear. It gives the impression that once you reach the independent phase everything stops’ (Expert 8)	Footprint sizes amended. Images changed. The steps were amended. The linear staircase shape was changed to a spiral.
Generality	The model’s use for a range of experiences; a general model will have wide applicability.	‘I am not convinced of the generality of the model. When I ask myself, ‘can this model be applied to a broad array of situations?’, I have to stop and think. The model is great in a context where adherence to radiation protection practice is not the norm. However, in areas where policies and guidelines are followed religiously, this model may not be fitting. The model cannot be seen as universal as this makes the assumption that there is a problem everywhere. If the researcher identified a radiation protection deficiency in Gauteng, or South Africa or wherever, then this model can be applied in this situation and is general to situations with similar deficiencies. So this model is general in practice areas where radiation protection is not practiced, but it is not general to areas where radiation protection is respected.’ (Expert 9)	The model is contextual to radiation protection non-compliance; therefore, generality is contextualised.
Accessibility	The model’s concepts emerging from empirical findings and achieving the model’s purpose.	All model experts found the model accessible: ‘The model can be regarded accessible as it is grounded in empirical evidence collected in previous phases of the study. It is also relatable to clinical experiences in the past and similar to other research findings in the South African context.’ (Expert 7)	None required.
Importance	The model’s practical and clinical value and assimilation into evidence-based practice.	All model experts found the model important: ‘I like the theory because it can apply radiographers working in all spheres of radiography [*within the radiography department, theatre and portable radiography*].’ (Expert 3)	None required.

Note: Please see the full experts details in Table 2.

### Limitations

The development of a model is a theoretical undertaking. In the current study, the model presented was evaluated by model experts who suggested changes. Once the changes were made to the model, the experts did not evaluate the model again. Therefore, the model would benefit from being evaluated post-amendment to ascertain whether the changes suggested by the experts were implemented accordingly. However, three examiners (separated from the expert reviews) reviewed the amended model. The model is yet to be implemented. The model was developed within the South African context with related nomenclature; however, the essence of the model can be applied worldwide when suboptimal radiation protection practices are evident.

## Conclusion

A model to facilitate radiation protection among radiographers premised on the central concept of ‘facilitating a transformative radiation protection environment’ sees the radiography manager and radiographer coming together to achieve optimal radiation protection in the radiography department through four phases. Optimal radiation protection prioritises patient care and human rights. The model has significance in addressing practice discord and, therefore, can be applied in medical imaging departments to ensure optimal service delivery.
